# Preliminary ^19^F-MRS Study of Tumor Cell Proliferation with 3′-deoxy-3′-fluorothymidine and Its Metabolite (FLT-MP)

**DOI:** 10.1155/2017/3981358

**Published:** 2017-09-26

**Authors:** In Ok Ko, Ki-Hye Jung, Mi Hyun Kim, Kyeung Jun Kang, Kyo Chul Lee, Kyeong Min Kim, Insup Noh, Yong Jin Lee, Sang Moo Lim, Jung Young Kim, Ji-Ae Park

**Affiliations:** ^1^Division of RI-Convergence Research, Korea Institute of Radiological & Medical Sciences, Seoul, Republic of Korea; ^2^Convergence Institute of Biomedical Engineering and Biomaterials, Seoul National University of Science & Technology, Seoul, Republic of Korea; ^3^Division of Medical Radiation Equipment, Korea Institute of Radiological & Medical Sciences, Seoul, Republic of Korea; ^4^Department of Chemical & Biomolecular Engineering, Seoul National University of Science & Technology, Seoul, Republic of Korea; ^5^Department of Nuclear Medicine, Korea Institute of Radiological & Medical Sciences, Seoul, Republic of Korea

## Abstract

The thymidine analogue 3′-deoxy-3′-[^18^F]fluorothymidine, or [^18^F]fluorothymidine ([^18^F]FLT), is used to measure tumor cell proliferation with positron emission tomography (PET) imaging technology in nuclear medicine. FLT is phosphorylated by thymidine kinase 1 (TK1) and then trapped inside cells; it is not incorporated into DNA. Imaging with ^18^F-radiolabeled FLT is a noninvasive technique to visualize cellular proliferation in tumors. However, it is difficult to distinguish between [^18^F]FLT and its metabolites by PET imaging, and quantification has not been attempted using current imaging methods. In this study, we successfully acquired* in vivo *^19^F spectra of natural or nonradioactive 3′-deoxy-3′-fluorothymidine ([^19^F]FLT) and its monophosphate metabolite (FLT-MP) in a tumor xenograft mouse model using 9.4T magnetic resonance imaging (MRI). This preliminary result demonstrates that ^19^F magnetic resonance spectroscopy (MRS) with FLT is suitable for the* in vivo* assessment of tumor aggressiveness and for early prediction of treatment response.

## 1. Introduction

**Figure 1:**
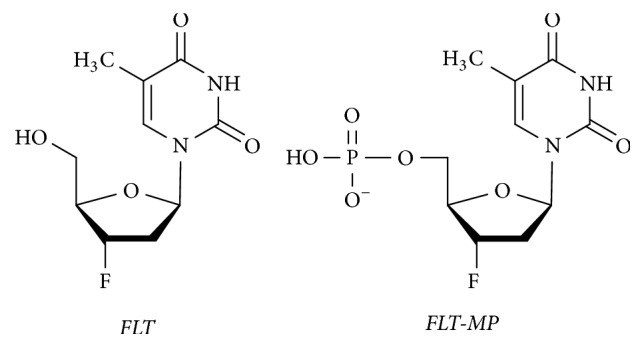
Chemical structures of FLT and FLT-MP.

Tumor cell proliferation is a useful prognostic indicator of tumor aggressiveness, and proliferation may be evaluated to monitor and predict the response to antitumor therapy. Tumor cells and tissues with a high proliferation rate require a high rate of DNA synthesis [1–5]. Radiolabeled thymidine analogues are standard biomarkers for DNA synthesis and are generally used in nuclear medicine. One thymidine analogue, [^11^C]-labeled thymidine, is well known as a radiotracer for positron emission tomography (PET) studies of tumor cell proliferation and DNA synthesis [6–9]. However, the short physical half-life (20 min) of [^11^C]-thymidine and its rapid biodegradation are practical limitations to its use [4, 10]. Consequently, the use of [^18^F]-labeled 3′-deoxy-3′-fluorothymidine ([^18^F]FLT) PET imaging to assess proliferation in various tumors has been reported in preclinical and clinical studies [11–13]. [^18^F]FLT in the cell is phosphorylated by the enzyme thymidine kinase 1 (TK1), producing [^18^F]FLT monophosphate ([^18^F]FLT-MP). [^18^F]FLT-MP can then be sequentially phosphorylated to form [^18^F]FLT diphosphate ([^18^F]FLT-DP) and [^18^F]FLT triphosphate ([^18^F]FLT-TP), which are metabolically trapped inside cells and are not incorporated into DNA (Figure 1) [14]. Li et al. demonstrated that metabolites of intracellular FLT during* in vitro* cell growth could be accurately measured with a liquid chromatography-tandem mass spectrometry (LC-MS/MS) assay [15]. However, this technique is considered a restrictive method, which is only used for* in vitro* drug screening at early stages.

^19^F magnetic resonance imaging (^19^F MRI) and spectroscopy (MRS) represent a promising* in vivo* quantitative imaging technique [16–18]. The nonradioactive isotope ^19^F has a 100% natural abundance with 83% sensitivity of ^1^H. The negligible background signal of endogenous ^19^F in biological systems provides an extremely high signal-to-nose ratio and exceptional sensitivity, making ^19^F MRI/MRS an ideal modality to monitor* in vivo* biochemical changes, in specific enzyme activity, cell tracking and migration, hypoxia, and quantitative neovascular responses [19, 20].

In this study, we monitored TK1 activity by quantifying FLT and FLT-MP* in vivo *using ^19^F MRI/MRS. Our aim was to develop and validate a suitable ^19^F MRI/MRS imaging biomarker for cellular proliferation in tumors.

## 2. Results and Discussion

**Figure 2:**
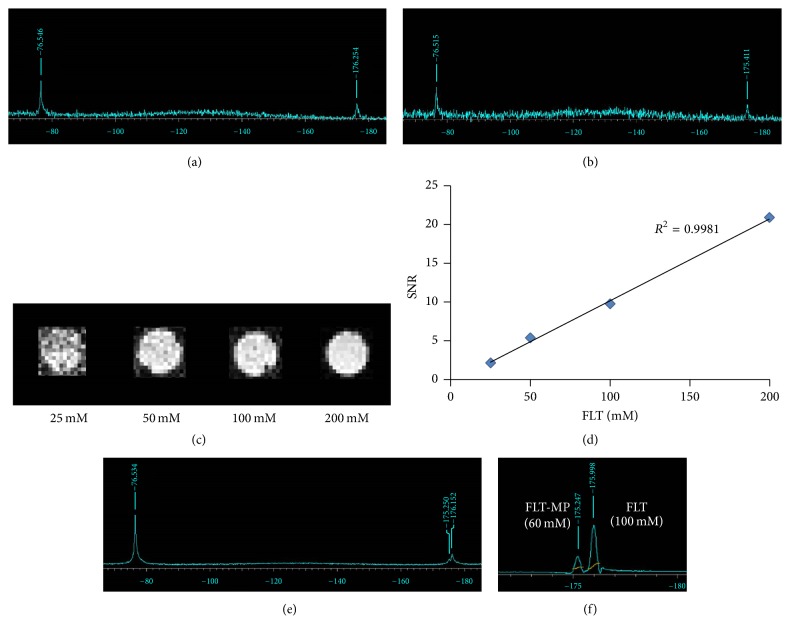
Typical coil-localized ^19^F spectra of (a) FLT and (b) FLT-MP containing TFA as a reference. (c) ^19^F MR images of phantoms containing 25, 50, 100, and 200 mM FLT. (d) Signal intensity in ^19^F MR images of FLT phantoms, as a function of FLT concentration (*R*^2^ = 0.998). (e) Typical coil-localized ^19^F spectrum of a phantom containing a mixture of FLT (100 mM) and FLT-MP (60 mM). (f) ^19^F MRS spectrum of a phantom containing a mixture of FLT (100 mM) and FLT-MP (60 mM). The area ratio of FLT (100 mM) to FLT-MP (60 mM) is approximately 100 to 60.

To detect the locations of FLT and FLT-MP, we investigated the ^19^F MRS of compounds containing TFA (−76.5 ppm) as a reference material. Figures 2(a) and 2(b) show that the spectra of FLT and FLT-MP were observed at −176.2 ppm and −175.4 ppm, respectively; the values were consistent with the NMR data (Figures S1 and S2, in Supplementary Material available online at https://doi.org/10.1155/2017/3981358).  Figure 2(c) shows ^19^F MR images of phantoms containing 25, 50, 100, and 200 mM of FLT, demonstrating that the signal intensity of ^19^F MR images corresponded with FLT concentration in phantoms (Figure 2(d)).  Figure 2(e) shows the spectrum of a mixture of FLT and FLT-MP, which were well separated at −176.2 ppm and −175.2 ppm, respectively.  Figure 2(f) shows the ^19^F MRS of the mixture; here, the former was FLT-MP and the latter was FLT. Because the area ratios of the spectra for the former and latter were approximately 60 and 100, respectively, the findings were consistent with the concentrations in the mixture of FLT-MP (60 mM) and FLT (100 mM).

We investigated whether the ^19^F NMR or ^19^F MRS signal of intracellular FLT-MP, produced as an FLT metabolite, could be detected* in vitro*. In the first group of cells that were not washed, both FLT and FLT-MP were clearly observed in the ^19^F NMR spectra, although the FLT-MP peak was very weak (Figure S3). However, the signal for FLT in the cells was very strong, and the concentration of FLT was 16.7 mM. In contrast, an FLT-MP peak in the first group of cells was not observed in the ^19^F MRS; only an FLT peak was observed (Figure S4).

**Figure 3:**
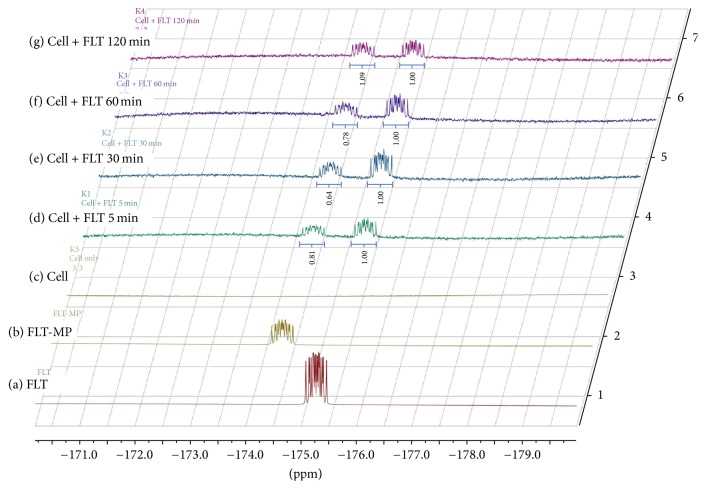
^19^F NMR spectra of MCF-7 cell suspensions treated with FLT (0.1 mg/1 × 10^7^ cells) as a function of time (d–g). The quantification in a relative ratio of FLT to FLT-MP was indicated. Typical spectra of (a) FLT (−175.4 ppm), (b) FLT-MP (−174.4 ppm), and (c) MCF-7 cells without FLT addition.

Figure 3 shows the ^19^F NMR spectra of washed cells in the second group as a function of time. Both intracellular FLT and FLT-MP were clearly observed at −175.2 ppm and −174.5 ppm, respectively. Because the extra FLT was washed out, the FLT signal exhibited a moderate level relative to the cells in the first group. Although the extra FLT was washed out, the presence of FLT demonstrated that FLT and its metabolites were reversible in the cell [2]. The amounts of intracellular FLT-MP and FLT were therefore inconsistent over time. A relative ratio of FLT to FLT-MP, here, demonstrated the on-going phosphorylation of different spectra in various tumors unlike PET technology.

No peak was observed in the ^19^F MRS signal for intracellular FLT-MP formed in the second group of cells because of its low concentration. These results demonstrated that a typical FLT concentration of 16.7 mM is required for* in vivo *detection by ^19^F MRS. As previous reports,* in vivo *^19^F MR imaging is generally used for the high concentration of 89 mM due to the low sensitivity of that [21].

**Figure 4:**
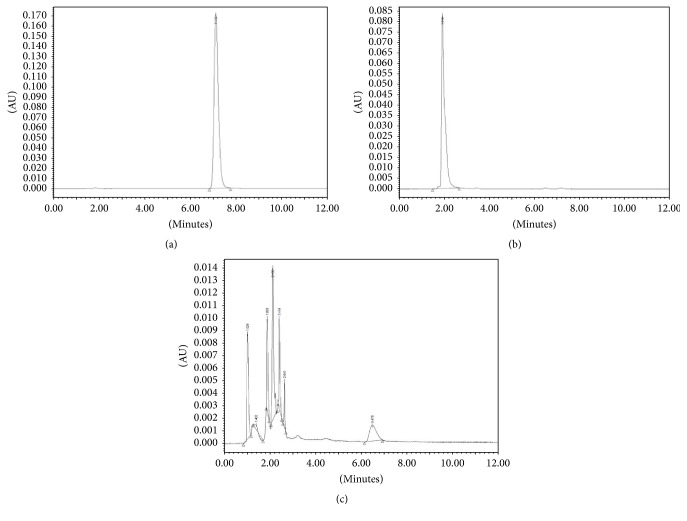
HPLC chromatograms of (a) FLT (Rt, 7.1 min), (b) FLT-MP (Rt, 2.0 min), and (c) MCF-7 cells treated with FLT.

To chemically confirm the accuracy of quantitation and metabolite detection by ^19^F MRS, an HPLC assay was performed.  Figure 4 shows HPLC chromatograms for FLT (Rt, 7.1 min) and FLT-MP (Rt, 2.0 min); the concentration was approximately 1 *μ*g/*μ*L. The* in vitro* HPLC chromatogram of the second group of cells demonstrated that FLT metabolism resulted in FLT-MP, FLT-DP, and FLT-TP production (Figure 4(c)).

**Figure 5:**
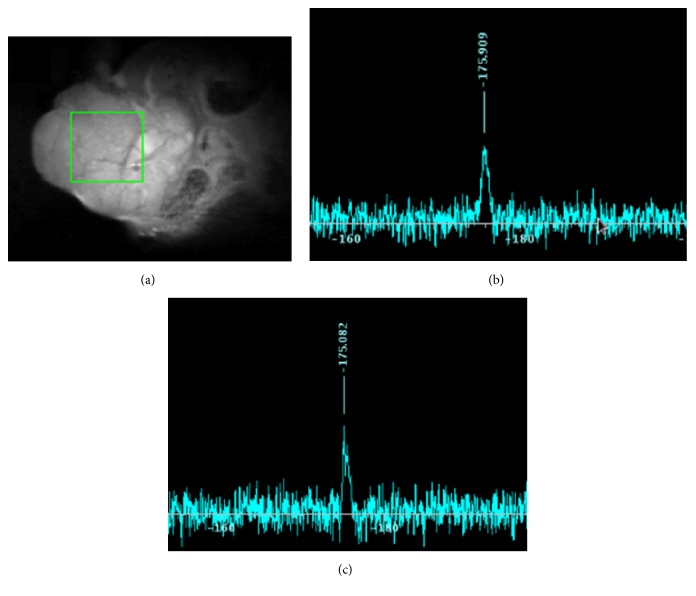
*In vivo* ^19^F MR spectrum in a mouse tumor model. FLT (200 mM, 100 *μ*L) was injected into tail veins. (a) Anatomical ^1^H MR images of the mouse were obtained using fast spin echo sequence with the voxel of interest in the tumor (5 × 5 × 5 mm^3^). Time-course of ^19^F MR spectra at (b) 25 min after injection (a.i.) (−175.9 ppm) and (c) 90 min a.i. (−175.08 ppm). ^19^F MRS data were acquired with Point-REsolved Spectroscopy (PRESS) using TR 3000 ms, TE 15 ms, averages 512, scan time 25 min.

We, then, investigated the* in vivo *^19^F MR signals for FLT and FLT-MP. More precisely, we aimed to observe that the appearance of the FLT-MP signal is caused by metabolism of FLT* in vivo*.  Figure 5(a) shows anatomical ^1^H MR images of a MCF-7 tumor in a mouse and the voxel of interest in the tumor for ^19^F MRS. In the 25 min after injection of FLT (200 mM, 100 *μ*L), a slight ^19^F signal was observed at −175.99 ppm (Figure 5(b)), corresponding with the location of FLT. After 90 min, the ^19^F signal was observed at −175.08 ppm (Figure 5(c)). Judging from the results of the phantom study, this signal represented FLT-MP despite being very weak.

Experimentally speaking, it was very difficult to accurately perform chemical shift imaging (CSI) of FLT and FLT-MP. Our studies, however, first detected a remarkable ^19^F MR signal in the tumors of living mice, thereby observing the metabolism of FLT by ^19^F MRS* in vivo*. Understanding the metabolism of FLT in a tumor-bearing mouse model may help us associate metabolism with PET data from [^18^F]FLT, a commonly used radiopharmaceutical in nuclear medicine; [^18^F]FLT is a good tracer of cell proliferation for assessment of tumor aggressiveness and early prediction of treatment response [12]. PET technology, high sensitivity, and the radiation of positron-emitting radioisotopes can easily permeate tissues, making PET a powerful molecular imaging modality to monitor the progression of cancer [22]. However, PET alone cannot readily distinguish between [^18^F]FLT and [^18^F]FLT-MP. Specifically, it is very difficult to simultaneously identify metabolites* in vivo* by kinetic analysis of FLT-PET images [8]. In that respect, our results show that ^19^F MRS is a noninvasive and practical way to identify biomolecules* in vivo*, including fluorine atoms; it may, thus, be utilized to complement other imaging tools, such as PET.

MRI/MRS is also a promising molecular imaging method for cancer theragnostics [23, 24]. For example, ^13^C MRI/MRS study of hyperpolarized [^13^C]pyruvate and its metabolite ([^13^C]lactate) could be recently used to measure early responses to therapy, and the utilization of metabolite levels has been studied in clinical practice [25–27]. The hyperpolarized ^13^C compounds, however, have restriction on the metabolism studies of DNA synthesis due to a time limit of hyperpolarization. The results of the present study, though preliminary, demonstrate that detection of [^19^F]FLT and its metabolite using ^19^F MRS might provide a novel avenue for cancer theragnostics.

## 3. Conclusion

In this study, FLT and its metabolite were measured for the first time in an* in vivo* mouse model using ^19^F MRS. This result showed that ^19^F MRS is suitable for the purpose of* in vivo* monitoring of specific drugs including radiopharmaceuticals and their metabolites. In addition, the findings of this study may support the clinical use of ^19^F MRI/MRS for the quantification and monitoring of the cellular proliferation in cancer and to assess the effectiveness of responses to therapy. Further studies are needed to improve the ^19^F MRS and CSI techniques for* in vivo* detection.

## 4. Materials and Methods

### 4.1. Chemicals

All reagents were purchased from commercial sources, and the following agents were FLT and FLT-MP (Research Center FutureChem Co., Ltd., Seoul, Korea) and trifluoroacetic acid (TFA) (Sigma-Aldrich, St. Louis, MO).

### 4.2. High-Performance Liquid Chromatography (HPLC)

The locations of compound were confirmed by analytical HPLC using an Atlantis C_18_ analytical column (5 *µ*m, 3.0 × 150 mm) with 10% EtOH in water (*v*/*v*) as the mobile phase at a flow rate of 0.4 mL/min. The retention times (*R*_*t*_) for FLT and FLT-MP were 7.1 min and 2 min, respectively.

### 4.3. Cell Culture

The MCF-7 human breast cancer cell line expressing the HSV-tk gene was maintained in RPMI-1640 medium supplemented with 10% FBS, 1% antibiotics, and 100 *μ*g/mL of G418 (Invitrogen). Cultures were maintained in a 37°C incubator with 5% CO_2_, and the medium was changed every 3 days.

For ^19^F MRS, MCF-7 cells were plated, and 5 × 10^7^ cells were suspended in 500 *μ*L of serum-free RPMI medium containing FLT (16.7 mM) before being incubated at 37°C for different time periods (5 min, 30 min, 60 min, and 120 min). The cells were divided into two groups. The first group of cells was not washed and was used for ^19^F NMR and ^19^F MRS. The second group of cells was washed three times with PBS, scraped from the plate, centrifuged at 1,000 rpm for 3 min, and then collected for use in ^19^F NMR, ^19^F MRS, and HPLC analyses.

For HPLC, the pellets were resuspended in PBS to a final volume of 1 mL and were then lysed by three cycles of freezing and thawing; the lysates were centrifuged at 14,000 rpm for 5 min at 4°C. The supernatant was used for HPLC analysis.

FLT and FLT-MP were extracted from the samples after growth for 5 min, 30 min, 60 min, or 120 min by three cycles of freezing and thawing. After centrifugation (14,000 rpm for 5 min at 4°C), the samples comprising a 90 : 10 mixture of supernatant: D_2_O, were placed in 5 mm nuclear magnetic resonance (NMR) tubes for data acquisition.

### 4.4. NMR

The ^19^F NMR measurements were conducted at 28°C on a Bruker 400-MHz NMR spectrometer, equipped with a 5-mm BBFO probe. The experimental parameters were as follows: pulse angle, 90° (18.32 *μ*sec); repetition rate, 1 sec; 172 K data set; 2,000 scans. All ^19^F data were processed using TopSpin and analyzed with MestReNova.

### 4.5. Animals

All animal experiments were conducted in compliance with the Guidelines for the Care and Use of Research Animals under protocols approved by the Korea Institute of Radiological and Medical Sciences (KIRAMS') Animal Studies Committee.

MCF-7 tumor cells (10^6^ cells/mL) suspended in RPMI serum-depletion media were inoculated into the subcutaneous tissue (sc) of female BALB/c nude mice (6 weeks, 20–25 g of body mass). The mice were anesthetized with 1.5% isoflurane. To monitor the formation of FLT-MP, ^1^H MRI and ^19^F MRS were performed after intravenous bolus injections of FLT (200 mM, 100 *μ*L).

### 4.6. MRI

All ^1^H MRI and ^19^F MRI/MRS data were acquired with a 9.4T animal MRI system and 20 mm surface coil (370–420 MHz) (Agilent Technologies, USA).

^1^H MR images were acquired with a fast spin echo sequence using the following settings: repetition time (TR) 2500 ms, echo time (TE) 25 ms, matrix 256 × 256, field of view (FOV) 5 × 5 cm^2^, slice thickness 2 mm, gap 0 mm, averages 2, and scan time 2 min 45 sec.

^19^F MR images of phantom were acquired with a gradient echo sequence using the following settings: TR 100 ms, TE 4.0 ms, matrix 64 × 64, FOV 5 × 5 cm^2^, slice thickness 2 mm, gap 0 mm, averages 1200, and scan time 2 h 8 min.

^19^F MRS of phantoms and in vivo data were acquired with Point-REsolved Spectroscopy (PRESS) using the following settings: TR 3000 ms, TE 15 ms, voxel size 5 × 5 × 5 mm^3^, averages 512, and scan time 25 min.

## Supplementary Material

Figure S1. ^19^F NMR spectrum of FLT.
Figure S2. ^19^F NMR spectrum of FLT-MP.
Figure S3. ^19^F NMR spectra of MCF-7 cells treated with FLT as a function of time. The formation of the FLT-MP signal caused by metabolism of FLT was observed at -174.5 ppm.
Figure S4. ^19^F MRS of MCF-7 cells treated with FLT.
