# Supplementary material for: Preliminary 19F-MRS Study of Tumor Cell Proliferation with 3′-deoxy-3′-fluorothymidine and Its Metabolite (FLT-MP)
Source: Contrast Media Mol Imaging. 2017 Sep 26;2017:3981358. doi: 10.1155/2017/3981358 (PMC5634584; doi:10.1155/2017/3981358)
Supplement: Supplementary file 1 — Figure S1. 19F NMR spectrum of FLT. Figure S2. 19F NMR spectrum of FLT-MP. Figure S3. 19F NMR spectra of MCF-7 cells treated with FLT as a function of time. The formation of the FLT-MP signal caused by metabolism of FLT was observed at -174.5 ppm. Figure S4. 19F MRS of MCF-7 cells treated with FLT. [file 3981358.f1.pdf]

Supplementary Information

Preliminary ^{19}F-MRS Study of Tumor Cell Proliferation with 3'-deoxy-3'-fluorothymidine (FLT) and its metabolite (FLT-MP)

In Ok Ko,[†, ‡] *Ki-Hye Jung,*[†] *Mi Hyun Kim,*[†] *Kyeung Jun Kang,*[†] *Kyo Chul Lee,*[†] *Kyeong Min Kim,*[‡] *Insup Noh,*[§] *Yong Jin Lee,*[†] *Sang Moo Lim,*[//] *Jung Young Kim,*[†, *] *Ji-Ae Park,*[†, *]

[†] Division of RI-Convergence Research, Korea Institute of Radiological & Medical Sciences, Seoul, Korea; [‡] Convergence Institute of Biomedical Eng. and Biomaterials, Seoul National Univ. of Sci. & Tech., Seoul, Korea; [‡] Division of Medical Radiation Equipment, Korea Institute of Radiological & Medical Sciences, Seoul, Korea; [§] Department of Chemical & Biomolecular Eng., Seoul National Univ. of Sci. & Tech., Seoul, Korea; [//] Department of Nuclear Medicine, Korea Institute of Radiological & Medical Science, Seoul, Korea.

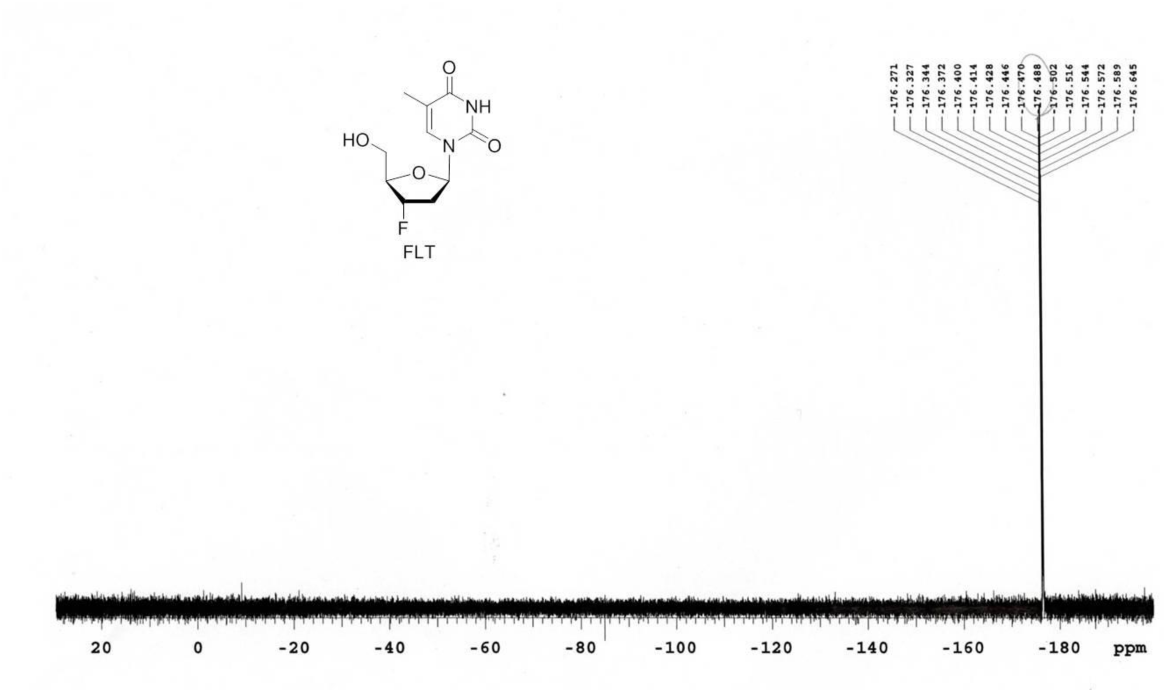

Figure S1. ^{19}F NMR spectrum of FLT

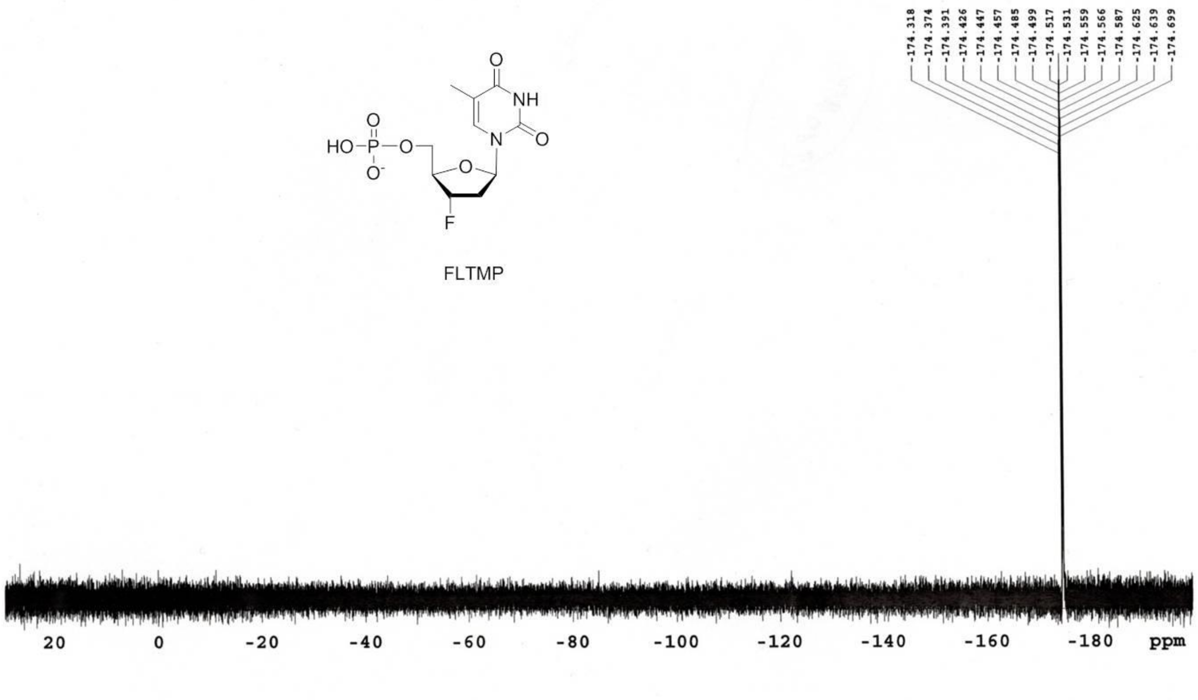

Figure S2. ^{19}F NMR spectrum of FLT-MP.

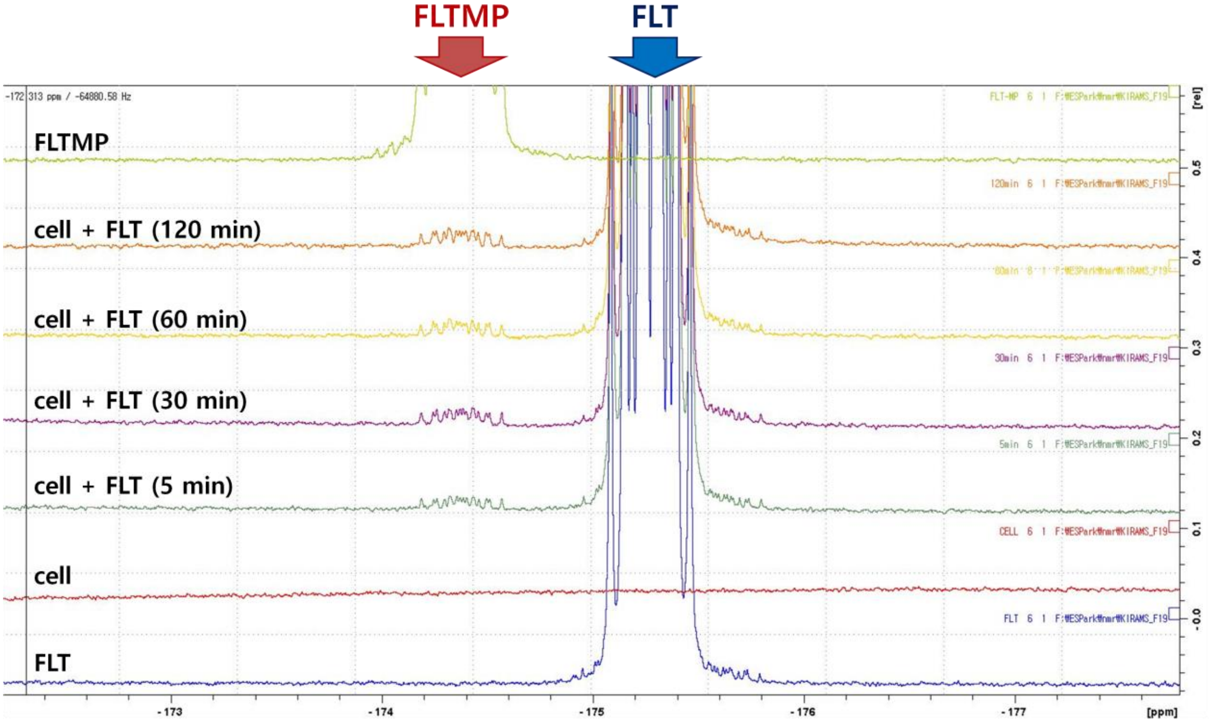

Figure S3. ^{19}F NMR spectra of MCF-7 cells treated with FLT as a function of time. The formation of the FLT-MP signal caused by metabolism of FLT was observed at -174.5 ppm.

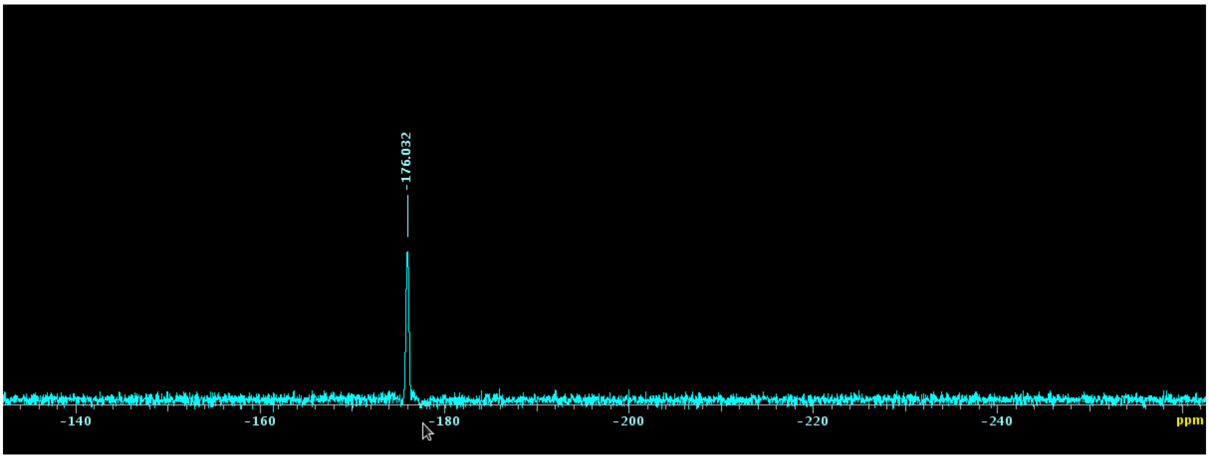

Figure S4. ^{19}F MRS of MCF-7 cells treated with FLT.
